# Colon Cancer-Specific Antigen-2 May Be Used as a Detecting and Prognostic Marker in Colorectal Cancer: A Preliminary Observation

**DOI:** 10.1371/journal.pone.0094252

**Published:** 2014-04-07

**Authors:** Gang Xue, Xiaojuan Wang, Yong Yang, Degui Liu, Ying Cheng, Jun Zhou, Yongkuan Cao

**Affiliations:** 1 Department of Breast and Thyroid Surgery, Chengdu Military General Hospital, Chengdu, P.R. China; 2 Department of Endocrinology, Chengdu Military General Hospital, Chengdu, P.R. China; 3 Department of General Surgery, Chengdu Military General Hospital, Chengdu, P.R. China; Vanderbilt University Medical Center, United States of America

## Abstract

**Background:**

A specific and sensitive serum marker for colorectal cancer (CRC) detection and surveillance is central to effective treatment. It was preliminarily reported that some nuclear matrix proteins may be served as a specific blood based marker for colon cancer. The objective of this study is to evaluate the value of serum CCSA-2 detection in diagnosis, prognostic estimation and surveillance for CRC.

**Method:**

Serum CCSA-2 protein was measured in 181 various patient populations and 20 healthy donors before surgery. For 106 CRC patients, it was also measured on day 7 after surgery. Among them, 49 CRC patients' CCSA-2 protein were measured during the follow-up period according to NCCN Guideline.

**Results:**

The serum CCSA-2 concentration in CRC patients was significantly higher than which in other patients and healthy individuals. Serum CCSA-2, at the cut-off point of 64.10 ng/mL, had a sensitivity of 98.10% and a specificity of 97.90% in separating CRC populations from all other individuals. The CCSA-2 assay was significantly more sensitive than CEA and CA19-9 assay in CRC detection. After surgery, the serum CCSA-2 level of CRC patients declined significantly, but it rebounded to a high level when recurrences occurred. The pre-operative serum CCSA-2 level in patients who had a relapse within the follow-up period was significantly higher than which in patients without relapse.

**Conclusions:**

Serum CCSA-2 not only may be a potential biomarker using in screening and surveillance of CRC, but also may be an independent prognostic marker for CRC patients. Further clinical trials need to be performed in a larger population of patients to ulteriorly confirm these results.

## Introduction

Colorectal cancer(CRC) is the third most common cause of cancer diagnosed in males and the second in females, it was estimated that more than 1.2 million new cases and about 600,000 deaths had been occurred worldwide in 2008 [1]. Each year there are 50 of every 100,000 persons were diagnosed and nearly 50,000 people were killed by CRC in United States [2].Survival is strongly related to stage at diagnosis, with five-year survival rates of 89.8% for localized cases(confined to the wall of the bowel), but only 67.7% for regional disease (disease with lymph node involvement) and 10.3% for distant metastatic patients [3]. In United State, despite advances in the management of CRC, the 5-year survival rate is only 62% on account of only 38% of patients are diagnosed when the cancers are localized to the bowel wall [4]. Screening and then diagnosis at an early stage can reduce the incidence of CRC in an advanced stage and hence mortality. An effective surveillance after treatment is also useful to find relapse timely, hence improve the patients' life quality and survival rate.

To date, the screening tests used in CRC can be divided into two groups: 1)Stool tests, mainly detect cancer, which include guaiac fecal occult blood testing (gFOBT), fecal immunochemical test (FIT) and testing stool for exfoliated DNA (sDNA); and 2) Structural examinations, can find cancer and advanced lesions as well as polyps, which include flexible sigmoidoscopy (FSIG), colonoscopy (CSPY), double-contrast barium enema (DCBE), and computed tomography colonography (CTC, also named virtual colonoscopy) [5], [6]. Each of these tests has some shortages. Some lack specificity, some are expensive or invasive, some cause bleeding or infection, and some need bowel preparation and cause bowel tear. So, a perfect test used in screening and surveillance for CRC should include the characteristics as follows: low invasive (or noninvasive), easy to perform, high sensitivity and specificity, safe and low costs.

Shortages mentioned above lead to low participants for CRC screening, however, compliance to a serum tests is likely be better than stool tests and structural examinations. Similar to prostate-specific antigen(PSA) blood test for prostate cancer, a novel serum-based biomarker called colon cancer-specific antigen-2(CCSA-2) was reported [7], which had been detected can be used as a potential marker for colon cancer detection with high sensitivity and specificity, but the value of serum CCSA-2 used in the aspects of prognostic estimation and surveillance after surgery for colorectal cancer was not studied. The relationship between CCSA-2 content and tumor stage, as well as nuclear grade was not reported yet. The purpose of this study was to investigate the value of serum CCSA-2 detection in diagnosis, prognostic estimation and surveillance after surgery for colorectal cancer.

## Materials and Methods

### Populations and samples

Serum samples were obtained from 181 patients and 20 healthy donors who signed the informed consent, and this study has been approved by Institutional Review Board of Health Ministry of Chengdu Military Area. Among these patients, 106 were diagnosed colorectal cancer(25 were colon cancer and 81 were rectal cancer) with pathohistological method. The other 75 patients(included 31 gastric cancer patients, 11 inguinal hernia patients, 8 acute appendicitis patients, 6 breast cancer patients and 19 colorectal benign disease patients) and 20 healthy donors constitute the control group(negative control, table 1). Each individual blood sample was collected with the vacuum blood collection tube (Becton Dickinson and Company, UK) 3 days before surgery(the sample from acute appendicitis patients were collected before emergency surgery). The samples were centrifuge at 4,000 rpm immediately, the supernatants were aliquoted in 1.5 ml tubes (Eppendorf, Germany) and were stored at −80°C conditions.

**Table 1:** The characteristics of participant population.

	Colorectal cancer**	Control***	*P* value
N	106	95	
Male	59	55	
Female	47	40	
Age(mean±SD)	58.62±11.93 years	56.31±17.25 years	0.37
Age(range)	23∼83years	18∼85years	
**Tumor stage(NCCN)** *			
I	26		
IIa	32		
IIIa	9		
IIIb	33		
IIIc	6		
**Tumor grade**			
G1	17		
G2	61		
G3	28		

*stage I: T1-2N0M0; stageIIA: T3N0M0; stageIIIA: T1-2N1/1cM0, T1N2aM0; stageIIIB: T3-4aN1/1cM0, T2-3N2aM0, T1-2N2bM0; stageIIIC: T4aN2aM0, T3-4aN2bM0, T4bN1-2M0.

**Colon cancer: 25, Rectal cancer: 81.

***Gastric cancer: 31, Breast cancer: 6, Inguinal hernia: 11, Acute appendicitis: 8, Colorectal benign disease: 19, Healthy donors: 20.

For all CRC patients, the serum samples were collected on day 7 after surgery. Among them, 49 patients have been finished 5 years follow-up, which serum samples were collected every 3–6 months during the follow-up period, referring to NCCN Clinical Practice Guidelines for colon cancer and rectal cancer. When recurrence was suspected, the colonoscopy and/or CT scan was performed to confirm if the regional recurrences or distant metastasis had occurred.

### Enzyme-linked immunosorbent assay(ELISA)

The serum CCSA-2 content was assayed according to the operational procedure using human CCSA-2 ELISA Kit(R&D Systems,USA). In brief, added 40 μl sample dilution and 10 μl serum sample to testing well in duplicates, then added 50 μl HRP-conjugate reagent, gently shaking, incubated for 60 min at 37°C. Removed the liquid and then washed the plate 5 times, added 50 μl Chromogen Solution A and B respectively to each well, mixed, incubated for 15 min at 37°C. Added 50 μl Stop Solution to each well and then measured the optical density(OD) at 450 nm(SpectraMax 190 Spectrophotometer, Molecular Devices Corporation, USA). Finally, calculated the sample CCSA-2 concentration according to standard curve.

### Compared the value of CCSA-2 with CEA and CA19-9 in detection of CRC

The serum CEA and CA19-9 levels were assayed using chemoluminescence method 3 days before surgery.

### Statistical analysis

The quantitative data were compiled as mean±standard deviation, and the qualitative data were complied as percentile.

To analyze differences among the pre-surgical groups, one-way analysis of variance (ANOVA) with the Tukey HSD post hoc test was performed. The colorectal cancer group was taken as reference. The same statistical method was used to analyze the differences among the different stages and different grades, it is also used to analyze the differences among pre-surgery, post-surgery and after recurrences of the patients who suffered regional relapsed and/or distant metastasis during the follow-up period. The differences between pre-surgery and post-surgery for all 106 CRC patients as well as negative controls were analyzed with ANOVA as well. The different value to detect CRC using serum CCSA-2 or CEA, CA19-9 was analyzed with Chi-square analysis. Receiver operator characteristic curve(ROC) was performed to evaluate the sensitivity and specificity of serum CCSA-2 to separate colorectal cancer patients from other individuals. The different pre-operative serum CCSA-2 levels in patients with recurrences and without recurrences after surgery was analyzed with T test.

All statistical analysis were performed using SPSS11.7 for Windows XP, statistical significance was assumed at *P*<0.05.

## Results

### The serum CCSA-2 content in different individuals and its diagnostic value for CRC

**Figure 1:**
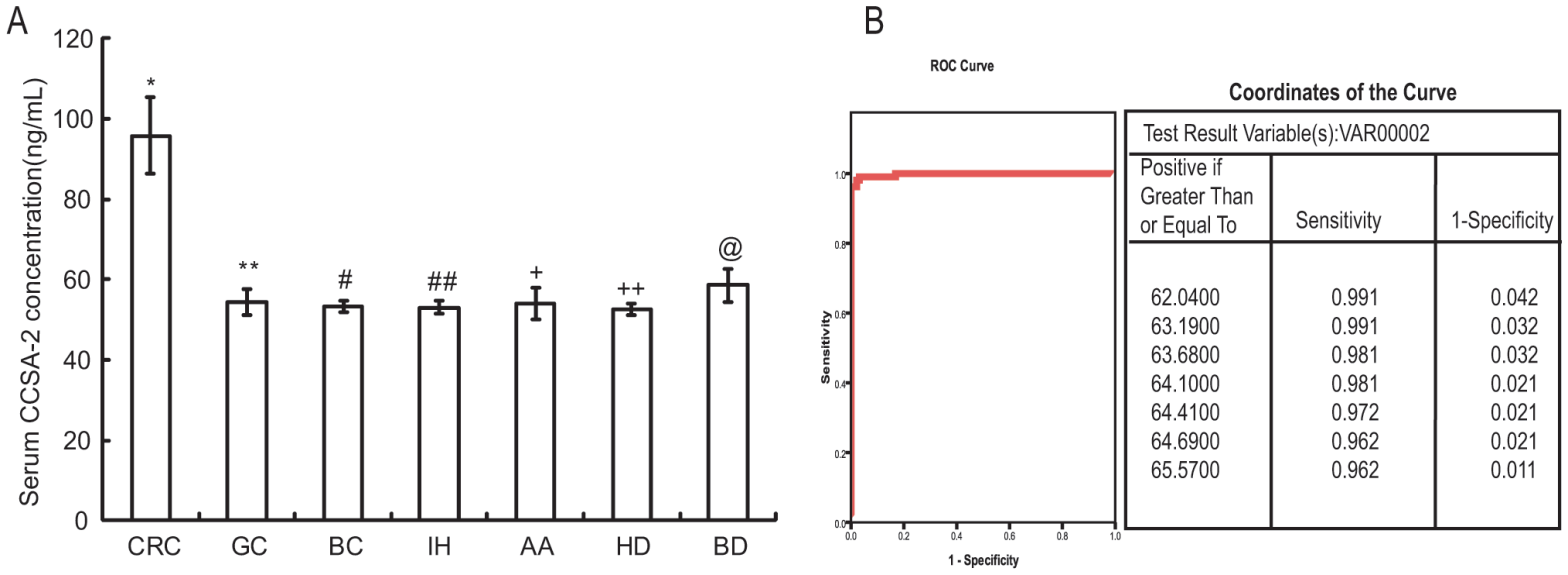
The use of serum CCSA-2 to detect CRC. (A) Comparison of serum CCSA-2 levels in colorectal cancer patients and other individual groups. *: the serum CCSA-2 concentration in CRC patients was significantly higher than each of the other individual groups(*P*<0.01); **,#,##,+,++,@: there were no statistical significances between other couple groups (*P*>0.05). (B) Receiver operator characteristic (ROC) curve for serum CCSA-2 in separating colorectal cancer patients and other individuals. CRC: colorectal cancer; GC: gastric cancer; BC: breast cancer; IH: inguinal hernia; AA: acute appendicitis; HD: healthy donor; BD: benign disease.

Using human CCSA-2 ELISA Kit to detect the content of CCSA-2 in serum from different populations. The average value of CCSA-2 in serum from 106 colorectal cancer patients was 95.78±9.57 ng/mL, whereas the average value for healthy individuals, gastric cancer patients, breast cancer patients, inguinal hernia patients, acute appendicitis patients, and colorectal benign disease patients(including adenoma, polyp and polyposis) were 52.51±1.37 ng/mL, 54.42±3.27 ng/mL, 53.29±1.37 ng/mL, 53.04±1.59 ng/mL, 53.97±4.09 ng/mL, and 58.45±4.11 ng/mL, respectively. Statistical analysis showed a highly significant difference in serum CCSA-2 concentration between the colorectal cancer patients and each of the other individual group(*F* = 265.37, *P*<0.01. figure 1), but there was no difference between other couple groups (*P*>0.05, figure 1). There were no differences of serum CCSA-2 content among the different tumor stages and different grades yet (*P*>0.05, table 2).

**Table 2:** CCSA-2 expression in different tumor stages and different tumor grades.

variation	N	CCSA-2(ng/ml)	statistic value	*P* value
**Dukes stage**				
A	28	94.25±8.11		
B	31	96.52±7.73		
C	47	93.68±6.85	*F* = 0.51	0.57
**TNM stage**				
I	26	93.21±8.01		
IIa	32	94.65±6.52		
IIIa	9	97.97±7.54		
IIIb	33	93.41±6.71		
IIIc	6	94.57±9.65	*F* = 0.42	0.71
**Nucleus grade**				
G1	17	93.89±7.56		
G2	61	95.21±8.52		
G3	28	96.12±9.12	*F* = 0.78	0.49

The receiver operator characteristic curve for serum CCSA-2 showed the serum CCSA-2 assay was highly accurate in separating colorectal cancer from all other individuals[area under the curve(AUC) was 0.998, 95% confidence interval, 95%CI, 0.00–1.00]. According to the ROC curve, the cut-off point of 64.10 ng/mL was selected, which had resulted in the optimal balance between sensitivity and specificity (figure 1).Using this cut-off point, the sensitivity of serum CCSA-2 to detect colorectal cancer was 98.10%, 104 of the 106 colorectal cancer patients were detected CCSA-2 in serum whose concentration were higher than 64.10 ng/mL; and the specificity was 97.90%, 93 of the other 95 individuals whose serum CCSA-2 concentration were lower than the cut-off point (table 3).

**Table 3:** Specificity/Sensitivity of Serum CCSA-2 Assay.

CCSA-2 level	>64.10 ng/mL	<64.10 ng/mL
CRC patients	104/106*	
Sensitivity	98.10%	
Other individuals		93/95**
Specificity		97.90%

*: the number of samples >64.10(ng/mL)/total samples; **: the number of samples <64.10(ng/mL)/total samples; CRC: colorectal cancer.

### The diagnostic value for CRC detection using serum CCSA-2 is higher than CEA or CA19-9

As a retrospective analysis, 94 patients among the 106 CRC patients were assayed the serum CEA levels, and 91 of the 106 CRC patients were assayed the serum CA19-9 levels using chemoluminescence method. In which 25 patients' CEA levels were higher than the upperbound of reference range (0–4 ng/mL), and 15 patients' serum CA19-9 were higher(range 0–40 IU/mL). Chi-square analysis demonstrated the serum CCSA-2 test was more sensitive than CEA or CA19-9 to detecting CRC(*χ* ^2^ = 111.29, *P*<0.01; *χ*2 = 136.42, *P*<0.01. table 4). There was no difference between CEA and CA19-9 (*χ* ^2^ = 2.79, *P* = 0.09).

**Table 4:** Compared the use of CCSA-2 with CEA or CA19-9 to detect colorectal cancer.

	total samples	positive samples	sensitivity
CCSA-2	106	104	98.10%
CEA	94	25	26.60%*
CA19-9	91	15	16.48%**

Chi-square analysis demonstrated serum CCSA-2 assay were more sensitive than CEA(*χ2 = 111.29, P<0.01) or CA19-9(**χ2 = 136.42, *P*<0.01) assay to detect colorectal cancer. There was no difference between CEA and CA19-9(*χ* ^2^ = 2.79, *P* = 0.09).

### The surveillance value of serum CCSA-2 detection in CRC patients

**Figure 2:**
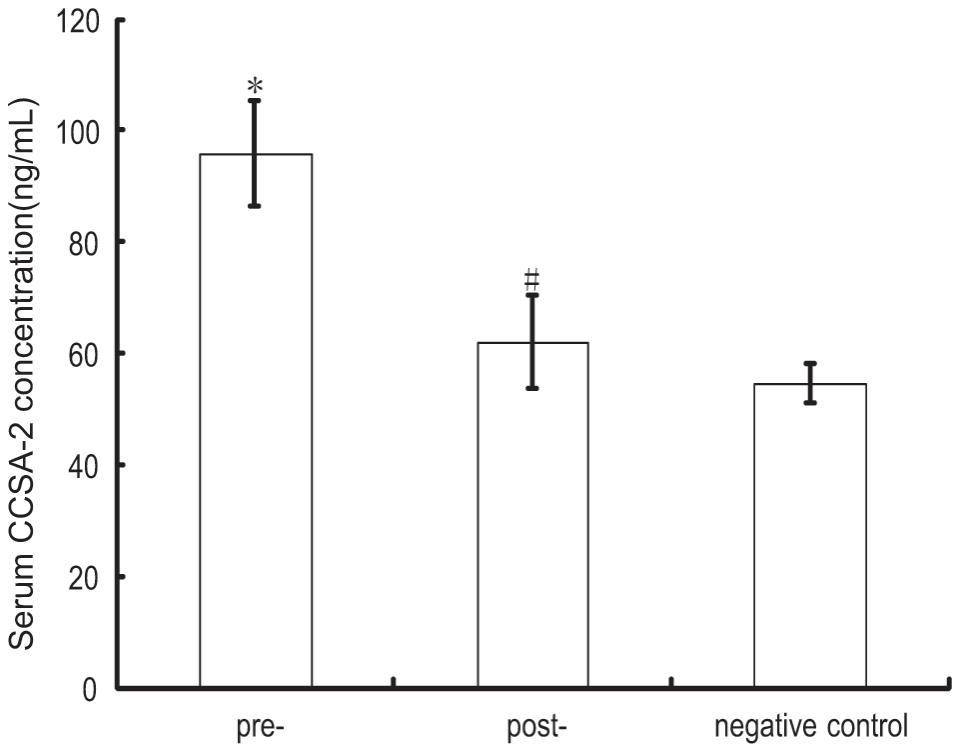
Pre- and post-operative levels of serum CCSA-2 in colorectal cancer patients. *,#: Statistic analysis showed the serum CCSA-2 concentration decreased significantly after surgery, but is still higher than the level of negative control (*F* = 827.57, *P*<0.001).

**Figure 3:**
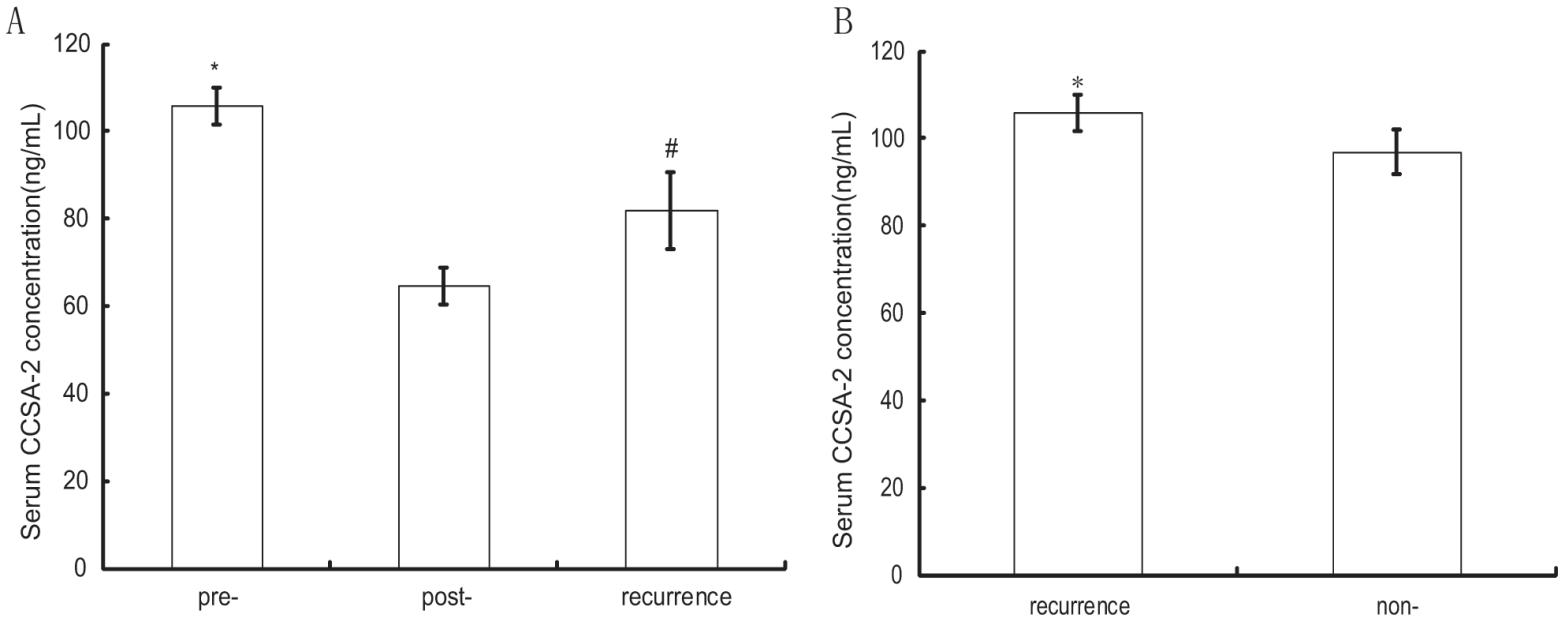
The use of serum CCSA-2 as a surveillance and prognostic marker for CRC. (A) Serum CCSA-2 levels in pre-operation, post-operation and after recurrences in patients who suffered relapse during the follow-up period. *:the serum CCSA-2 concentration in pre-operation was significantly higher than each of the other individual groups (*P*<0.01); #: the serum CCSA-2 levels after recurrences was higher than that in post-operation (*P*<0.01). (B) Comparison of pre-operative serum CCSA-2 levels in patients with recurrences and without recurrences during the follow-up period after surgery. *: Statistic analysis showed the pre-operative serum CCSA-2 concentration in patients with recurrences was significantly higher than those in patients without recurrences after surgery (*t* = 6.18, *P*<0.001).

On the seventh day after operation, the serum CCSA-2 level in CRC patients decreased significantly to 62.03±8.39 ng/mL, but it was still higher than the level of negative control, which was 54.56±3.61 ng/mL(figure 2, *F* = 827.57, *P*<0.001). Among the 49 CRC patients, 13 suffered from regional relapse and/or hepatic metastasis during the follow-up period, the recurrence period ranged from 5 to 47 months(average 20.38±15.50 months) after surgery. And their serum CCSA-2 level rebounded to 81.84±8.70 ng/mL, which significantly higher than the cut-off point and the level on the seventh day after surgery, but lower than pre-operative level (figure 3, *P*<0.01). Further analysis revealed that the CCSA-2 level before surgery in patients who suffered recurrences during the follow-up period was higher than that in patients without recurrences (105.75±4.11 ng/mL vs 96.93±5.15 ng/mL, figure 3, *t* = 6.18, *P*<0.001).

## Discussion

Recent trends show the CRC incidence and mortality were decline, which is attributed to early detection through screening and improved treatment [8]. This reveals that early diagnosis and early detection of recurrences are important strategies for effective treatment of CRC. CRC screening is very effective to prevent the occurrence of many cases and to detect CRC in early and curable stage, and regular surveillance after surgery is important for the detection of recurrences without delay, hence improve patients' life quality and prolong their lifespan. At present, only a few adults has received regular age-eligible and risk-eligible screening, although it was demonstrated that the reductions of incidence and mortality in CRC had been largely attributed to early detection of invasive disease and adenomatous polyps [9], [10]. So, if a greater proportion of appropriate adults received regular screening, a greater incidence and mortality reductions in the near term could be achieved [11].

Although there are a range of options can be chose for screening and surveillance, but there is little consensus or guideline about which kind of screening method is the best one, in the aspect of sensitivity and specificity, patient acceptance, invasiveness, safety, and cost-effectiveness, limit current options. Each test has unique advantages, cost-effectiveness, limitations and risks [12], [13]. A positive stool blood test, gFOBT or FIT, should be followed up with colonoscopy to determine if the cancer or advanced polyps is present. The cost of stool test-sDNA is much higher than the other stool tests, and the test frequency is uncertain. DCBE requires extensive colonic preparation, patients may feel uncomfortable, and it lacks opportunity for biopsy or polypectomy [14]. FSIG can only examine lower half of the colon lumen and some patients complain about periprocedural discomfort. The sensitivity of virtual colonoscopy (VC) for large adenomas and CRC appears to be high but for small adenomas is low, its detecting rates vary by centre and there is a steep learning curve. Colonoscopy is considered as a gold standard for CRC diagnosis with a sensitivity of 97% and a specificity of 98%, but it is associated with high cost, patient discomfort, various complications, and operator-skill dependence; it also requires dietary preparation, bowel cleaning, sometimes needs sedation and a chaperone.

A perfect CRC screening approach must have high sensitivity and specificity. Also it would be better to have the advantages including non-invasion or minimal invasion, safety, low cost, convenience, and so on. To this end, the serum marker examination may accomplish these criteria. Now, some serum markers such as CEA, CA19-9 (carbohydrate antigen19-9), CA242 (carbohydrate antigen242), TPS (tissue polypeptide specific antigen) have been used in CRC diagnosis and treatment, but each of them possesses neither high sensitivity nor high specificity to enable use them as screening markers for CRC in the asymptomatic population. Actually,these serum markers were more used as recurrent and prognostic markers in clinical practice [15], [16], [17], [18], [19].

Seeking for a specific and sensitive serum marker which can be used to detect CRC at an early stage is central to effective treatment. Nuclear matrix protein (NMP) has been identified as an oncological “fingerprint” for some certain cancers, such as bladder, renal, and prostate cancers. Brunagel and his colleagues had identified colon cancer-specific nuclear matrix proteins that were present in cancer tissue, but not found in normal adjacent tissue or in the normal colon tissue [20], [21], [22]. The investigators then reported that two colorectal cancer-specific proteins-colorectal cancer-specific antigen (CCSA)-3 and CCSA-4 had the sensitivity of 100%, the specificity of 82% and 91% respectively to detect CRC [22]. Their researches also revealed that the CCSA-2 had overall sensitivity of 97.3% and specificity of 78.4% in separating individuals with CRC and advanced adenomas from normal, non-advanced adenomas and hyperplastic populations [7]. Although the use of NMP, especially serum CCSA-2,3,4 in detection of CRC was studied, but the use of serum CCSA-2,3,4 in prognostic estimation and surveillance after surgery for CRC was not studied, the relationship between CCSA-2 content and tumor stages, grades was not reported yet.

In this study, we detected serum CCSA-2 levels in different individuals using ELISA method. Surprisingly, at a cut-off point of 64.10 ng/mL, the serum CCSA-2 concentration has the sensitivity of 98.10% and the specificity of 97.90% in separating individuals with CRC from other participant population, which consists of gastric cancer, inguinal hernia, acute appendicitis, breast cancer patients, healthy donors and colorectal benign disease patients. This result is more inspiring than the result which was reported previously by Brunagel and his colleagues. The serum CCSA-2 detection with ELISA method is of low injury, high safety and high cost-effectiveness, high sensitivity and specificity; and only 1 mL blood sample is needed. It would be the best serum marker for CRC screening. Using serum CCSA-2 test in appropriate population may improve early detecting rate of CRC, and it is likely to be of paramount importance to ultimate curing of the vast majority of CRC patients.

To observe the changes of serum CCSA-2 value after the removal of the colorectal cancer by surgery, samples were obtained from all 106 CRC patients on day 7 after curative surgery. Compared to the pre-operative level, the serum CCSA-2 concentration after surgery decreased significantly, which was lower than the diagnostic cut-off point of 64.10 ng/mL. But it was still higher than the level of negative controls (containing other patients and healthy donors). We speculate it may be related to the incomplete lymph node dissection or some micrometastatic lesions existence which can not be detected by using routine examination when the serum samples are collected, it may also be related to the existence of circulating tumor cells. This phenomenon revealed that serum CCSA-2 detection may be used as a marker to evaluate the thoroughness of surgery.

Among the 106 CRC patients, 49 were finished 5 years follow-up. Unfortunately, 13 patients suffered regional recurrences and/or hepatic metastasis, their serum CCSA-2 expression were up-regulated again, even exceeding the level on day 7 after surgery and the diagnostic cut-off point. It will be a useful approach for early detection of recurrences of CRC after surgery, hence improve patients lifespan and life quality. This surveillance method may also reduce patients' cost and boost their compliance to accept regular examination during the follow-up period, avoid unnecessary invasive investigations along with their severe complications.

There was no correlation of the serum CCSA-2 expression and the tumor stages (NCCN TNM stage or Dukes stage) and we did not observe a correlation between the serum CCSA-2 level and the tumor cell grades. This may reveal that CCSA-2 is only related to tumorigenesis but not related to tumor progress in CRC. However, the pre-operative serum CCSA-2 level in the patients who suffered recurrences were significantly higher than those in patients without recurrences during the follow-up period after surgery. This result revealed that the serum CCSA-2 may be used as a prognostic marker for colorectal cancer, just like the Her-2 gene amplification used as a hazard prognostic marker for breast cancer, the higher serum CCSA-2 expression may indicate a worse prognosis and a higher risk of recurrences after surgery for CRC patients.

Although Knychalski B et al [23] revealed CCSA-2 as a single tumor marker had a low diagnostic value in CRC because of low sensitivity and specificity when compared to CEA, our study showed that CCSA-2 was more sensitivity than CEA or CA19-9 to detect CRC (98.10% vs 26.60% and 16.48%, respectively), the difference may be derived from different sample size, race, region, or different methods of assay and statistic analysis.

In conclusion, serum CCSA-2 detection may be used as a useful approach for CRC screening and surveillance, and its expression is only related to tumorigenesis. Serum CCSA-2 may also be used as a prognostic molecular marker for CRC. This method is likely to be cheaper, safer, more acceptable to patients; and hopefully, it will provide a uniform approach to CRC screening and surveillance. Although our study achieved a surprising result, it was just a preliminary clinical observation based on small sample size. Further clinical trials need to be performed, for evaluation of the sensitivity and specificity in independent validation studies in a larger population of patients.
